# A Population Based Study of the Genetic Association between Catecholamine Gene Variants and Spontaneous Low-Frequency Fluctuations in Reaction Time

**DOI:** 10.1371/journal.pone.0126461

**Published:** 2015-05-15

**Authors:** Jojanneke A. Bastiaansen, Tarrant D. R. Cummins, Harriëtte Riese, Arie M. van Roon, Ilja M. Nolte, Albertine J. Oldehinkel, Mark A. Bellgrove

**Affiliations:** 1 Interdisciplinary Center Psychopathology and Emotion regulation, Department of Psychiatry, University of Groningen, University Medical Center Groningen, Groningen, The Netherlands; 2 School of Psychological Sciences, Monash University, Melbourne, VIC, Australia; 3 Department of Epidemiology, University of Groningen, University Medical Center Groningen, Groningen, The Netherlands; 4 Department of Vascular Medicine, University Medical Center Groningen, Groningen, The Netherlands; Sichuan University, CHINA

## Abstract

The catecholamines dopamine and noradrenaline have been implicated in spontaneous low-frequency fluctuations in reaction time, which are associated with attention deficit hyperactivity disorder (ADHD) and subclinical attentional problems. The molecular genetic substrates of these behavioral phenotypes, which reflect frequency ranges of intrinsic neuronal oscillations (Slow-4: 0.027-0.073 Hz; Slow-5: 0.010-0.027 Hz), have not yet been investigated. In this study, we performed regression analyses with an additive model to examine associations between low-frequency fluctuations in reaction time during a sustained attention task and genetic markers across 23 autosomal catecholamine genes in a large young adult population cohort (*n* = 964), which yielded greater than 80% power to detect a small effect size (*f^2^* = 0.02) and 100% power to detect a small/medium effect size (*f^2^* = 0.15). At significance levels corrected for multiple comparisons, none of the gene variants were associated with the magnitude of low-frequency fluctuations. Given the study’s strong statistical power and dense coverage of the catecholamine genes, this either indicates that associations between low-frequency fluctuation measures and catecholamine gene variants are absent or that they are of very small effect size. Nominally significant associations were observed between variations in the alpha-2A adrenergic receptor gene (*ADRA2A*) and the Slow-5 band. This is in line with previous reports of an association between *ADRA2A* gene variants and general reaction time variability during response selection tasks, but the specific association of these gene variants and low-frequency fluctuations requires further confirmation. Pharmacological challenge studies could in the future provide convergent evidence for the noradrenergic modulation of both general and time sensitive measures of intra-individual variability in reaction time.

## Introduction

Intra-subject variability in reaction time (RT) contains specific temporal components of relevance for different forms of psychopathology [1]. A greater magnitude of low-frequency fluctuations in RT (< 0.1 Hz) has been associated with ADHD and subclinical attentional problems [1–6]. This suggests that an inability to modulate low-frequency RT fluctuations may underlie behavioral symptoms such as difficulty sustaining attention [1,2]. The default mode interference hypothesis [7] proposes that slow periodic fluctuations in behavioral performance are caused by an ineffective shift from a default mode to an active processing mode during cognitive challenges. The inability to modulate low-frequency RT fluctuations could be caused by a catecholamine deficiency [2]. Catecholamines dopamine (DA) and noradrenaline (NA) are critical for the ability to sustain attention [8,9] and recent studies have shown that they modulate deactivation of the default mode network (DMN) and its functional coupling with task-related networks (e.g. [10–14]). The question then arises: do alterations in the regulation of catecholamine signaling modulate the strength of low-frequency RT fluctuations?

Gene variants that influence the functioning of catecholamines could throw light on this relationship. A plausible candidate is the functional val158met single-nucleotide polymorphism (SNP) on the catechol-O-methyltransferase (*COMT*) gene [15,16], which affects baseline DA levels [17–19]. The *COMT*/val158met SNP may be involved in low-frequency RT fluctuations, because its effect on cognition seems specific for tasks that require the maintenance of task-relevant representations [20–22], and it has been associated with altered functional connectivity within and between DMN and task-relevant regions [13,23,24]. The relationship between low-frequency RT fluctuations and catecholamine gene variants has not yet been addressed. Cummins *et al* [25] did recently show that variations in the alpha-2A adrenergic receptor gene (*ADRA2A*) are associated with *general* intra-individual RT variability using an association approach focused on the catecholamine system. Moreover, RT variability mediated the relationship between the *ADRA2A* SNP rs1800544 and self-reported ADHD symptoms.

Here, we used a similar association approach to study the molecular genetics of low-frequency (i.e. time sensitive) RT fluctuations. We administered a sustained attention RT task in a large young adult population cohort to investigate associations between the power in two low-frequency bands reminiscent of the DMN’s rhythm (Slow-4: 0.027–0.073 Hz, Slow-5: 0.010–0.027 Hz [3,26]) and catecholamine gene variants. We explored SNPs and a variable number tandem repeat (VNTR) polymorphism from 23 autosomal catecholamine genes, with *COMT*/val158met and *ADRA2A* gene variants as a priori candidates.

## Materials and Methods

### Study sample

We used neuropsychological data from the fourth wave (T4) and genetic data of the TRacking Adolescents’ Individual Lives Survey (TRAILS), a large representative prospective population cohort study from the North of the Netherlands. The baseline sample comprised a diverse sample of 2230 10–12 year-olds of predominantly Dutch ethnicity (89.7%), of whom 1881 (83.4%) participated at T4 and 1515 (77.4% of the total T4 TRAILS sample) successfully completed a sustained attention task (criteria are described in [1]). Genotypic data were available for 1095 of these participants, of whom one of each sibling pair and participants from non-Dutch ancestry were excluded (*n* = 87). An additional 44 participants were excluded because they used medication targeting catecholaminergic systems, which could affect our behavioral measures (see below). The final sample for this study therefore included 964 individuals (mean age: 19.0 ± 0.6 years, 53.3% females). The selected participants were slightly younger (mean age = 19.0 ± 0.6 years) than the unselected T4 participants (*n* = 790, mean age = 19.2 ± 0.6 years, *t*(1752) = 7.4, *p* < .001), but similar in male-female ratio (*t*(1752) = 1.0, *p* = .31). The TRAILS study was approved by the Dutch Central Committee on Research Involving Human Subjects (CCMO, no. NL22114.042.08). Written informed consent was obtained from both the parents and the participants. For more details on the sampling procedure and methods see [27–29].

### Medication exclusion

Medication use was coded according to the Anatomical Therapeutic Chemical (ATC) classification system of the WHO Collaborating Centre for Drug Statistics Methodology (http://www.whocc.no). Participants were excluded if they used medication targeting catecholaminergic systems, that is, cardiovascular medication based on beta blocking agents (C07A) such as propranolol (adrenergic receptor blocker), drugs for functional gastrointestinal disorders (A03) such as domperidone (dopamine receptor antagonist), adrenergic respiratory medication (R03A) such as salbutamol (adrenergic receptor agonist), psychostimulants (N06B) such as methylphenidate (dopamine reuptake inhibitor), antipsychotics (N05A) such as haloperidol (mainly dopamine receptor antagonists), and the sex hormone cabergoline (a dopamine receptor agonist).

### Neurocognitive task

The Sustained Attention Dot (SAD) patterns task was part of a test battery of five tasks assessing neurocognitive functioning taken from the Amsterdam Neuropsychological Tasks (ANT) battery [30]. During the SAD task, participants had to respond as quickly as possible to patterns with 3, 4 or 5 dots by pressing the mouse button with their dominant hand (‘yes’ response) when there were 4 dots, and pressing the mouse button with their non-dominant hand (‘no’ response) when there were 3 or 5 dots. The three types of dot patterns occurred with equal frequency and were randomly presented across 50 series of 12 trials without breaks. A dot pattern disappeared immediately after a response had been given (valid response window: 200–6000 ms), entailing that the task pace was largely determined by the participant’s behavior. Each stimulus was followed by a fixed post response interval of 250 ms.

### Frequency domain analysis

Detailed procedures concerning data preparation, frequency domain definitions, and frequency domain analysis have been described previously [1]. In brief, the RT data were: 1) normalized by a natural logarithmic transformation (ln) with a displacement parameter of 150 ms; and 2) individually adjusted for the average reaction time for each stimulus type (i.e., 3, 4, or 5 dots). Power spectral analysis was applied to these adjusted logarithmic RT series (alnRT) based on fast Fourier Transform using CARSPAN [31]. Power was calculated for neurophysiologically defined oscillation bands Slow-4 (0.027–0.073 Hz) and Slow-5 (0.010–0.027 Hz) [3,26] as DMN-oscillations have generally been related to frequencies below 0.1 Hz (e.g. [32,33]). Note that power is a relative measure; it indicates the percentage of the total variance the Slow-4 and Slow-5 bands account for in a particular subject. Higher values are indicative of an amplification at these power spectra (i.e., a greater magnitude of these low-frequency RT fluctuations).

### DNA extraction and Genotyping

Blood samples or buccal swabs (Cytobrush) were collected at T3. DNA extraction, using the manual salting out procedure [34], and genotyping of all SNPs were performed at the Department of Genetics, University Medical Center Groningen, The Netherlands. Genotyping was executed on the Golden Gate Illumina BeadStation 500 platform (Illumina Inc., San Diego, CA, USA) according to the manufacturer’s protocol. Genotype data clustering was performed in BeadStudio 3.0 (Illumina Inc., San Diego, CA, USA). Concordance between DNA replicates showed a 100% genotyping accuracy. Data cleaning was in line with standard procedures [35]. In addition to this candidate gene genotyping, genome-wide genotyping was performed using the Illumina HumanCytoSNP12 v2 beadchip assay (Illumina, Inc; San Diego, CA, USA). Genotypes were called with the Illumina GenomeStudio software package (Illumina, Inc). Details on genotyping and quality control have been given elsewhere [36]. Next, imputation was carried out with Impute v2 [37] using the 1000 Genomes (release March 2012) as reference panel.

For the *DRD4* VNTR polymorphism, the 48 base pair repeat sequence located in exon 3 was determined by direct analysis on an automated capillary sequencer (ABI3730, Applied Biosystems, Nieuwerkerk a/d IJssel, The Netherlands) using standard conditions at the CCKL quality-certified Research lab for Multifactorial Diseases within the Human Genetics department of the Radboud University Nijmegen Medical Centre (Nijmegen, The Netherlands). Three percent blanks as well as duplicates between plates were included for quality controls. Call rate and DNA replicate consistency were 98% and 100%, respectively. Genotypic data for the *DRD4* VNTR were coded with 0, 1, and 2 to represent the number of copies of the 7 repeat allele.

### Genetic variant selection

Genetic variants were selected according to the catecholamine system-wide association approach developed by Cummins and colleagues [25,38]. In brief, SNPs were selected from 23 autosomal catecholamine genes, namely those that are known to be involved in synthesis, degradation, transport and receptor signaling of DA and/or NA (as identified in the KEGG, [http://www.genome.jp/kegg/pathway.html] and Gene Ontology [http://www.geneontology.org]) metabolic pathway databases). Note that the *ANKK1* gene was included in the set of autosomal catecholamine genes, because SNPs within this gene are in strong linkage disequilibrium (LD) with SNPs of *DRD2* [39]. Haplotype tagging SNPs were identified to provide 5′–3′ coverage of each gene using the HapMap project database [40]. We favored the inclusion of SNPs as tags if they had been shown to modify the risk for ADHD, were at exon—intron boundaries (300bp) or mapped to within 5kb of the genes of interest including the 5′ and 3′ untranslated (UTR) regions, which may have regulatory roles. Only tagging SNPs (r^2^ ≥ 0.8) with a minor allele frequency of > 0.1 were selected (see [25] for SNP selection criteria). This process yielded dense coverage of the 23 autosomal catecholamine genes with 152 tagging SNPs. In addition, the 48 base pair VNTR mapped to exon 3 of the *DRD4* gene was included.

This selection of SNPs/VNTRs was extracted from the TRAILS database. We included rs4680 of the *COMT* gene and SNPs for the *ADRA2C* and *PNMT* genes that failed during genotyping in the original papers [25,38]. However, two previously studied VNTRs in the *SLC6A3* gene [25,38] were not genotyped in the TRAILS dataset and hence could not be included in this study.

### Genetic association analyses

Initial correlation analyses indicated that while age was not significantly associated with the Slow-4 and Slow-5 measures, gender approached significant association at uncorrected levels with Slow-4 (*r* = .06, *p* = .056) and was significantly associated at corrected significance levels with Slow-5 (*r* = .16, *p*<.001). Thus, gender was included as a covariate in a single step Monte-Carlo permutation method that tested for genetic associations with Slow-4 and Slow-5 via an additive regression model. Permutation methods are considered the gold standard when conducting association analyses, because they provide unbiased control for type 1 error and maintain statistical power while correcting for multiple comparisons. As densely typed SNP sets show complex patterns of correlation (LD) arising from DNA recombination, our permutation method corrected for correlated samples (SNP data) to give a critical value of *α* = .00042 for examining the association between a cognitive measure and any genetic marker (see [25] for a full description of this method). We then corrected for the number of cognitive variables examined (2; Slow-4, Slow-5) to give a final critical value, of *α* = .00021 (.00042/2), *α*_FWE_ = .05. With this permutation method that stringently corrects for both genotypic and phenotypic measures, our sample size of 964 participants yielded greater than 80% power to detect a small effect size (*f* ^*2*^ = 0.02) and 100% power to detect a small/medium effect size (*f* ^*2*^ = 0.15). We also present results that are significant at the uncorrected significance level for our a priori candidates (*COMT*/val158met and *ADRA2A* gene variants), and note that we have 100% power to detect associations of small effect size at this level. Power calculations were performed using G*Power 3.1 [41].

## Results

### Genotyping

149 SNPs remained after a quality control process (S1 Table). SNPs that were excluded from analyses were: rs11568324, rs3730287 and rs62388321 (*SLC6A2*, *ADRA1A* and *DRD1* genes, respectively), as the former SNP had a MAF <.05 in our sample while the latter two SNPs were homozygous for the major allele. All of the remaining SNPs were in Hardy Weinberg Equilibrium (at *p*_*critical*_ = .001).

### Genetic association analyses

**Fig 1:**
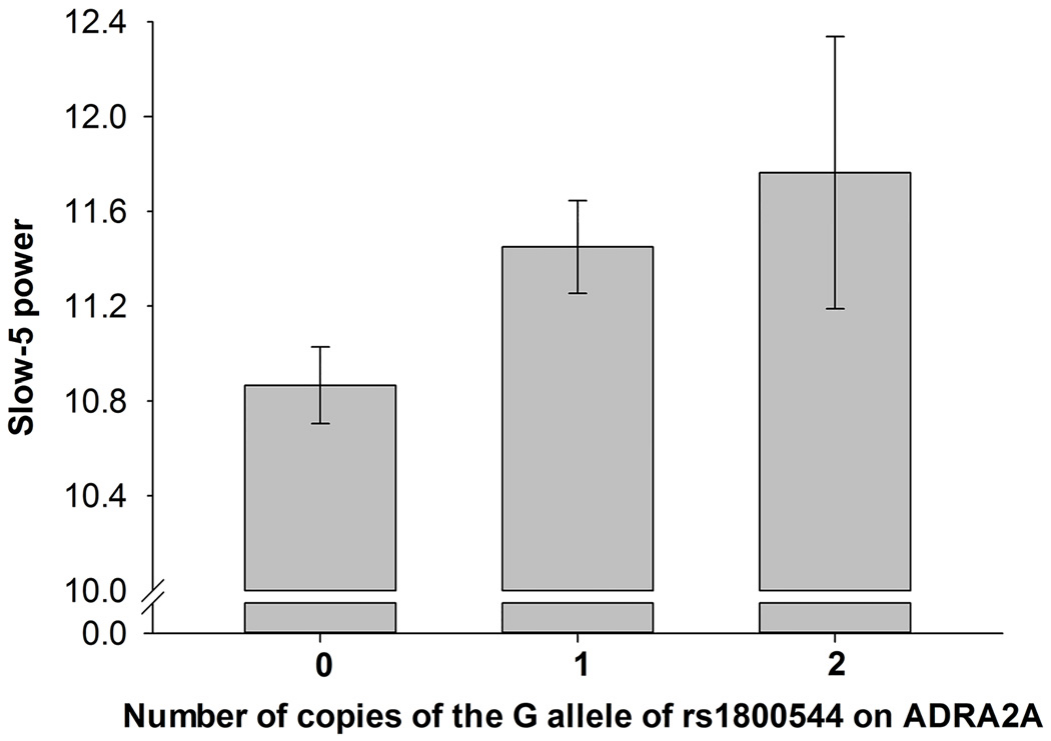
The relationship between genotypic variation at *ADRA2A* rs1800544 and the low-frequency RT fluctuation measure Slow-5. Relative power in the Slow-5 band (%) increased with each copy of the G allele.

The *COMT*/val158met SNP was not significantly associated with either RT variability measure (Slow-4, Slow-5) at corrected or uncorrected levels (Table 1). The *ADRA2A* SNP rs1800544 showed a significant association at the uncorrected level with the low-frequency RT fluctuation measure Slow-5 (*p*_uncorrected_ = .007, *r*_sp_ = .089). There was an additive increase in relative Slow-5 power with each copy of the G allele of rs1800544 (Fig 1). Additionally, each of the three other *ADRA2A* SNPs (rs521674, rs11195419, rs602618) showed association at nominal (uncorrected) significance (Table 1). Although a number of other SNPs distributed across the 23 autosomal catecholamine genes showed nominally significant associations, none (including the *ADRA2A* SNPs) survived correction for multiple comparisons (S1 Table).

**Table 1:** The influence of COMT/val158met and ADRA2A gene variants on low-frequency fluctuation measures Slow-4 and Slow-5.

Chromosome/Gene	Position	Marker ID	MAF	Slow-4	Slow-5
22/COMT	19951271	rs4680	0.43	.856	.112
10/ADRA2A	112835590	rs521674	0.26	.907	.013*
10/ADRA2A	112836503	rs1800544	0.25	.933	.007*
10/ADRA2A	112839368	rs11195419	0.10	.326	.023*
10/ADRA2A	112843085	rs602618	0.26	.904	.017*

Position: chromosomal position under build GRCh37.p13. MAF: minor allele frequency. Slow 4 and Slow 5: p perm value.

* = achieved nominal (uncorrected) significance,

*α* = .05 (but not significant when corrected for the number of cognitive measures and genetic markers examined, corrected critical value *α* = .00021).

## Discussion

Catecholamine systems have been implicated in low-frequency RT fluctuations, which are associated with attentional problems. In this study, we examined associations between low-frequency RT fluctuation measures (Slow-4, Slow-5) and genetic markers. The results were clear-cut: at corrected significance levels, none of the gene variants were associated with the magnitude of low-frequency RT fluctuations. Despite our a priori hypothesis, there were no associations between the *COMT*/val158met SNP and the Slow-4 or Slow-5 measures at either the corrected or uncorrected significance levels. Associations were observed between all *ADRA2A* gene variants and Slow-5, but only at the uncorrected level. Critically, our tagging SNP design provided dense coverage of 23 autosomal catecholamine genes, which entails that any association signal generated from an unselected catecholamine SNP would be well represented by its proxy (tag) SNP. In addition, our large sample size provided sufficient statistical power to detect associations of small effect size after correction for multiple comparisons. Thus, the fact that none of the SNP associations survived correction for multiple comparisons either indicates that associations between low-frequency RT fluctuation measures and catecholamine SNPs are not to be found or that they are of very small effect size in this general population sample.

The fact that catecholamine gene variants were not associated with low-frequency RT fluctuations during a sustained attention task does not exclude the possibility that different results might have been obtained with a different task. However, the long SAD task with its limited cognitive demand seems very suited to induce attentional lapses. Both children and adults with ADHD have shown impairments on the task, which can be improved by methylphenidate (e.g. [42–44]). Moreover, low-frequency RT fluctuations documented during this task have been linked to attentional problems in a general population sample [1]. Further, the task is qualitatively very similar to other cognitive paradigms such as the Sustained Attention to Response Task (SART), which have been used to document differences between ADHD cases and controls in low-frequency RT fluctuations [4,5]. While some studies have found a stronger link with attentional problems for the Slow-4 band rather than the Slow-5 band or higher-frequency bands [1, 3], the specificity is still a matter of debate [45].

It is also possible that measures of intra-subject variability may not be invariant across cognitive tasks. Through principal component analysis on general variability measures (i.e. intra-individual coefficients of variation, ICVs) across different cognitive tasks, Cummins and colleagues [25] recently found two variability components. Tasks that, similar to our SAD task, required subjects to select one response from among competing response alternatives loaded strongly on the component termed the response selection variability factor [25]. Individual differences in this factor were predicted by variation in the *ADRA2A* gene, and the factor mediated the relationship between the *ADRA2A* SNP rs1800544 and self-reported ADHD symptoms. In this context, it is noteworthy that we observed a small but significant association at the uncorrected significance level for all of the *ADRA2A* SNPs under study here and individual differences in the Slow-5 RT fluctuation measure. Although *ADRA2A* seems implicated in RT fluctuations, the directionality and specificity of effects requires further investigation. In contrast to additive decreases in (absolute) ICV scores in the previous paper, we found that *relative* power in Slow-5 increased with each copy of the G-allele (rs1800544) and C-allele (rs602618). However, higher relative power in a frequency band does not preclude lower overall variance. The strong LD between rs1800544 lying in the promoter region and rs602618 lying in the 3′ untranslated region (*r* ^*2*^ = .93, *D'* = .97) of the *ADRA2A* gene suggests that these results may reflect a functional variant that lies between these SNPs or within a greater haplotype block.

One may speculate about the significance of the association between *ADRA2A* gene variants and intra-subject variability as assessed here using low-frequency fluctuations and in [25] using ICV. It is well-established that pharmacological modulation of the pre-synaptic α2A autoreceptor using low dose clonidine reduces noradrenergic release and induces attentional lapses [46]. These effects can be reversed using the α2A antagonist idazoxan [46]. That methylphenidate is able to reduce attentional lapses and stabilize intra-subject variability in both ADHD and non-ADHD populations [2, 47–49] may be understood in terms of its well-documented effect on alpha2a receptors in prefrontal cortex [50]. Thus, methylphenidate may selectively engage the α2A receptor and so promote an optimized prefrontal cortex signal-to-noise ratio, which stabilizes fluctuations in top-down control of attention that may contribute to intra-subject variability. The associations observed between *ADRA2A* gene variants and Slow-5 further suggest that stimulation of the α2A receptor may play a role in slow periodic fluctuations in behavioral performance that are reminiscent of the DMN’s intrinsic rhythm (e.g. [51]). Whereas some studies suggest that low-frequency oscillations in the Slow-5 band might be most robust in default mode structures and Slow-4 oscillations might be strongest in the basal ganglia [51–53], the exact brain-behavior relationship of the frequency bands still requires clarification. The specific relationship between the α2A receptor, RT fluctuations, and the DMN, could be tested using selective α2A agents to observe both modulations of intra-subject variability (ICV or Slow-4 and Slow-5) and the intrinsic rhythm of the DMN.

## Conclusions

In sum, we did not find strong evidence for associations between catecholamine gene variants and low-frequency RT fluctuations. Our findings are in line with previous reports of an association between *ADRA2A* gene variants and RT variability during response selection tasks, but the specific association of these gene variants and low-frequency fluctuations requires further confirmation. Studies are now needed that integrate behavioral and neurophysiological measures across a range of cognitive domains to elucidate the functional meaning of the different frequency bands. Moreover, pharmacological challenge studies using receptor specific α2A agents could provide important convergent evidence for the noradrenergic modulation of both general (ICV) and time sensitive (e.g., Slow-5) measures of intra-individual variability.

## Supporting Information

S1 Dataset (SAV)

S1 Table The influence of common catecholamine gene variants on the low-frequency RT fluctuation measures of Slow-4 and Slow-5. (DOCX)
