# Supplementary material for: A Population Based Study of the Genetic Association between Catecholamine Gene Variants and Spontaneous Low-Frequency Fluctuations in Reaction Time
Source: PLoS One. 2015 May 15;10(5):e0126461. doi: 10.1371/journal.pone.0126461 (PMC4433112; doi:10.1371/journal.pone.0126461)
Supplement: S1 Table — (DOCX) [file pone.0126461.s002.docx]

**Table S1.** **The influence of common catecholamine gene variants on the low-frequency RT fluctuation measures of Slow-4 and Slow-5.**

| **Chromosome/Gene** | **Position** | **Marker ID** | **MAF** | **Slow-4** | **Slow-5** |
| --- | --- | --- | --- | --- | --- |
| 2/ADRA2B | 96777168 | rs2312955 | 0.38 | 0.176 | 0.992 |
| 3/DRD3 | 113847283 | rs3732790 | 0.41 | 0.365 | 0.039* |
| 3/DRD3 | 113858201 | rs2134655 | 0.29 | 0.906 | 0.444 |
| 3/DRD3 | 113862887 | rs963468 | 0.4 | 0.485 | 0.048* |
| 3/DRD3 | 113867862 | rs324036 | 0.11 | 0.634 | 0.051 |
| 3/DRD3 | 113876275 | rs167771 | 0.15 | 0.497 | 0.082 |
| 3/DRD3 | 113881623 | rs324029 | 0.27 | 0.227 | 0.642 |
| 3/DRD3 | 113885135 | rs11706283 | 0.08 | 0.902 | 0.927 |
| 3/DRD3 | 113887068 | rs7633291 | 0.19 | 0.573 | 0.687 |
| 3/DRD3 | 113890815 | rs6280 | 0.31 | 0.426 | 0.177 |
| 3/DRD3 | 113900220 | rs9825563 | 0.29 | 0.919 | 0.671 |
| 3/DRD3 | 113912270 | rs17605608 | 0.19 | 0.203 | 0.694 |
| 3/DRD3 | 113918633 | rs6762200 | 0.33 | 0.466 | 0.494 |
| 4/ADRA2C | 3770952 | rs7678463 | 0.17 | 0.596 | 0.999 |
| 4/DRD5 | 9779580 | rs10033951 | 0.32 | 0.232 | 0.395 |
| 4/DRD5 | 9785265 | rs1967550 | 0.36 | 0.503 | 0.743 |
| 5/SLC6A3 | 1416142 | rs40358 | 0.15 | 0.571 | 0.220 |
| 5/SLC6A3 | 1418374 | rs37020 | 0.41 | 0.432 | 0.909 |
| 5/SLC6A3 | 1428135 | rs10053602 | 0.23 | 0.334 | 0.617 |
| 5/SLC6A3 | 1428514 | rs393795 | 0.19 | 0.044* | 0.386 |
| 5/SLC6A3 | 1430616 | rs11737901 | 0.38 | 0.339 | 0.536 |
| 5/SLC6A3 | 1432825 | rs460000 | 0.19 | 0.047* | 0.560 |
| 5/ADRB2 | 148206440 | rs1042713 | 0.35 | 0.314 | 0.859 |
| 5/ADRB2 | 148206473 | rs1042714 | 0.44 | 0.962 | 0.867 |
| 5/ADRA1B | 159347481 | rs2030373 | 0.24 | 0.799 | 0.556 |
| 5/ADRA1B | 159348443 | rs6884105 | 0.34 | 0.605 | 0.469 |
| 5/ADRA1B | 159351460 | rs756275 | 0.08 | 0.344 | 0.064 |
| 5/ADRA1B | 159360485 | rs6892282 | 0.42 | 0.708 | 0.700 |
| 5/ADRA1B | 159367114 | rs6888306 | 0.21 | 0.500 | 0.525 |
| 5/ADRA1B | 159367275 | rs13162302 | 0.15 | 0.886 | 0.845 |
| 5/ADRA1B | 159369429 | rs7737796 | 0.37 | 0.709 | 0.736 |
| 5/ADRA1B | 159381080 | rs17057305 | 0.16 | 0.992 | 0.867 |
| 5/ADRA1B | 159383271 | rs12653825 | 0.31 | 0.669 | 0.415 |
| 5/ADRA1B | 159390877 | rs952037 | 0.3 | 0.301 | 0.413 |
| 5/ADRA1B | 159391811 | rs11953285 | 0.1 | 0.018* | 0.961 |
| 5/DRD1 | 174867899 | rs4867798 | 0.32 | 0.912 | 0.175 |
| 5/DRD1 | 174868700 | rs686 | 0.37 | 0.309 | 0.103 |
| 5/DRD1 | 174870196 | rs5326 | 0.16 | 0.319 | 0.313 |
| 5/DRD1 | 174870902 | rs265981 | 0.37 | 0.327 | 0.110 |
| 7/DDC | 50525420 | rs4947510 | 0.31 | 0.915 | 0.477 |
| 7/DDC | 50526219 | rs11575553 | 0.11 | 0.084 | 0.218 |
| 7/DDC | 50538589 | rs11238131 | 0.31 | 0.693 | 0.945 |
| 7/DDC | 50545593 | rs12718529 | 0.44 | 0.630 | 0.880 |
| 7/DDC | 50549880 | rs17634958 | 0.13 | 0.121 | 0.764 |
| 7/DDC | 50560255 | rs6592952 | 0.44 | 0.625 | 0.781 |
| 7/DDC | 50564204 | rs3807566 | 0.45 | 0.156 | 0.316 |
| 7/DDC | 50570136 | rs880028 | 0.22 | 0.098 | 0.113 |
| 7/DDC | 50574012 | rs1817074 | 0.35 | 0.014* | 0.027* |
| 7/DDC | 50591070 | rs12535064 | 0.24 | 0.205 | 0.044* |
| 7/DDC | 50596864 | rs3735273 | 0.23 | 0.231 | 0.380 |
| 7/DDC | 50597301 | rs11575334 | 0.48 | 0.956 | 0.337 |
| 7/DDC | 50620229 | rs2329340 | 0.35 | 0.356 | 0.514 |
| 7/DDC | 50623451 | rs10249420 | 0.24 | 0.142 | 0.175 |
| 7/DDC | 50631862 | rs7807335 | 0.24 | 0.138 | 0.191 |
| 7/DDC | 50634976 | rs12666409 | 0.24 | 0.118 | 0.224 |
| 8/ADRA1A | 26610152 | rs1157690 | 0.15 | 0.283 | 0.841 |
| 8/ADRA1A | 26611846 | rs17055923 | 0.21 | 0.496 | 0.190 |
| 8/ADRA1A | 26617904 | rs4732641 | 0.35 | 0.748 | 0.188 |
| 8/ADRA1A | 26628028 | rs1048101 | 0.43 | 0.390 | 0.059 |
| 8/ADRA1A | 26636906 | rs60593443 | 0.13 | 0.213 | 0.480 |
| 8/ADRA1A | 26692367 | rs472151 | 0.45 | 0.553 | 0.256 |
| 8/ADRA1A | 26695255 | rs2322333 | 0.42 | 0.477 | 0.851 |
| 8/ADRA1A | 26695617 | rs10503800 | 0.3 | 0.230 | 0.872 |
| 8/ADRA1A | 26697057 | rs574647 | 0.28 | 0.420 | 0.131 |
| 8/ADRA1A | 26697365 | rs577366 | 0.25 | 0.257 | 0.225 |
| 8/ADRA1A | 26698471 | rs472865 | 0.13 | 0.675 | 0.482 |
| 8/ADRA1A | 26703750 | rs523816 | 0.33 | 0.143 | 0.721 |
| 8/ADRA1A | 26710290 | rs486354 | 0.28 | 0.248 | 0.151 |
| 8/ADRA1A | 26711214 | rs2046186 | 0.21 | 0.454 | 0.744 |
| 8/ADRA1A | 26713707 | rs580739 | 0.26 | 0.071 | 0.251 |
| 8/ADRA1A | 26716205 | rs13274396 | 0.23 | 0.221 | 0.717 |
| 8/ADRA1A | 26717123 | rs563097 | 0.13 | 0.573 | 0.370 |
| 8/ADRA1A | 26721296 | rs528257 | 0.39 | 0.999 | 0.260 |
| 8/ADRA1A | 26724326 | rs3808585 | 0.3 | 0.755 | 0.132 |
| 8/ADRB3 | 37820093 | rs9694197 | 0.06 | 0.035* | 0.467 |
| 8/ADRB3 | 37821486 | rs4998 | 0.06 | 0.090 | 0.977 |
| 8/ADRB3 | 37823798 | rs4994 | 0.06 | 0.090 | 0.977 |
| 9/DBH | 136500515 | rs1611115 | 0.2 | 0.601 | 0.712 |
| 9/DBH | 136501941 | rs2797849 | 0.35 | 0.634 | 0.060 |
| 9/DBH | 136507742 | rs1548364 | 0.45 | 0.350 | 0.003* |
| 9/DBH | 136509634 | rs2519152 | 0.47 | 0.672 | 0.027* |
| 9/DBH | 136512515 | rs2797853 | 0.34 | 0.716 | 0.418 |
| 9/DBH | 136514668 | rs6479643 | 0.41 | 0.052 | 0.256 |
| 9/DBH | 136518097 | rs77905 | 0.48 | 0.154 | 0.682 |
| 9/DBH | 136519590 | rs10761412 | 0.39 | 0.378 | 0.694 |
| 9/DBH | 136522274 | rs6271 | 0.06 | 0.473 | 0.137 |
| 9/DBH | 136523669 | rs129882 | 0.22 | 0.912 | 0.103 |
| 10/ADRA2A | 112835590 | rs521674 | 0.26 | 0.907 | 0.013* |
| 10/ADRA2A | 112836503 | rs1800544 | 0.25 | 0.933 | 0.007* |
| 10/ADRA2A | 112839368 | rs11195419 | 0.1 | 0.326 | 0.023* |
| 10/ADRA2A | 112843085 | rs602618 | 0.26 | 0.904 | 0.017* |
| 10/ADRB1 | 115805056 | rs1801253 | 0.27 | 0.534 | 0.606 |
| 10/ADRB1 | 115807016 | rs3813720 | 0.37 | 0.115 | 0.900 |
| 11/DRD4 | 636399 | rs3758653 | 0.18 | 0.957 | 0.670 |
| 11/DRD4 | 637622 | rs752306 | 0.05 | 0.310 | 0.304 |
| 11/DRD4 | 641191 | rs11246226 | 0.46 | 0.444 | 0.432 |
| 11/DRD4 | 643348 | rs7395429 | 0.45 | 0.520 | 0.356 |
| 11/TH | 2184848 | rs3842727 | 0.31 | 0.070 | 0.041* |
| 11/TH | 2186335 | rs2070762 | 0.47 | 0.110 | 0.256 |
| 11/TH | 2190519 | rs7483056 | 0.43 | 0.277 | 0.155 |
| 11/TH | 2190951 | rs6356 | 0.37 | 0.517 | 0.157 |
| 11/TH | 2192798 | rs10840489 | 0.11 | 0.191 | 0.962 |
| 11/ANKK1 | 113258065 | rs10891545 | 0.14 | 0.422 | 0.847 |
| 11/ANKK1 | 113264272 | rs17115439 | 0.35 | 0.470 | 0.133 |
| 11/ANKK1 | 113266821 | rs7118900 | 0.19 | 0.775 | 0.481 |
| 11/ANKK1 | 113270160 | rs2734849 | 0.48 | 0.369 | 0.070 |
| 11/ANKK1 | 113270828 | rs1800497 | 0.2 | 0.517 | 0.821 |
| 11/DRD2 | 113281073 | rs6279 | 0.32 | 0.598 | 0.078 |
| 11/DRD2 | 113283688 | rs1076560 | 0.15 | 0.327 | 0.607 |
| 11/DRD2 | 113285536 | rs2283265 | 0.15 | 0.345 | 0.561 |
| 11/DRD2 | 113286878 | rs2440390 | 0.14 | 0.747 | 0.139 |
| 11/DRD2 | 113301069 | rs2471851 | 0.15 | 0.279 | 0.501 |
| 11/DRD2 | 113305314 | rs12364051 | 0.4 | 0.397 | 0.038* |
| 11/DRD2 | 113308902 | rs17115583 | 0.11 | 0.641 | 0.184 |
| 11/DRD2 | 113317973 | rs4245146 | 0.47 | 0.934 | 0.932 |
| 11/DRD2 | 113321796 | rs4460839 | 0.11 | 0.802 | 0.419 |
| 11/DRD2 | 113329774 | rs7131056 | 0.45 | 0.424 | 0.813 |
| 11/DRD2 | 113335717 | rs4350392 | 0.13 | 0.558 | 0.967 |
| 16/SLC6A2 | 55689087 | rs2397771 | 0.36 | 0.266 | 0.626 |
| 16/SLC6A2 | 55695106 | rs3785143 | 0.09 | 0.734 | 0.257 |
| 16/SLC6A2 | 55706391 | rs36024 | 0.39 | 0.122 | 0.950 |
| 16/SLC6A2 | 55711950 | rs36021 | 0.43 | 0.824 | 0.207 |
| 16/SLC6A2 | 55718818 | rs36017 | 0.44 | 0.466 | 0.268 |
| 16/SLC6A2 | 55722850 | rs1345429 | 0.45 | 0.545 | 0.485 |
| 16/SLC6A2 | 55729836 | rs3785157 | 0.37 | 0.292 | 0.467 |
| 16/SLC6A2 | 55730124 | rs5568 | 0.35 | 0.492 | 0.300 |
| 16/SLC6A2 | 55731735 | rs8047672 | 0.18 | 0.884 | 0.805 |
| 16/SLC6A2 | 55732620 | rs36009 | 0.08 | 0.290 | 0.542 |
| 16/SLC6A2 | 55733589 | rs1800887 | 0.22 | 0.486 | 0.182 |
| 16/SLC6A2 | 55735350 | rs8049681 | 0.15 | 0.531 | 0.307 |
| 16/SLC6A2 | 55735912 | rs2242447 | 0.31 | 0.284 | 0.633 |
| 16/SLC6A2 | 55737691 | rs7194256 | 0.15 | 0.554 | 0.296 |
| 16/SLC6A2 | 55741297 | rs9930182 | 0.21 | 0.524 | 0.161 |
| 17/PNMT | 37824545 | rs876493 | 0.43 | 0.584 | 0.531 |
| 20/ADRA1D | 4201984 | rs709024 | 0.4 | 0.830 | 0.381 |
| 20/ADRA1D | 4205060 | rs3787441 | 0.24 | 0.642 | 0.713 |
| 20/ADRA1D | 4210448 | rs8183794 | 0.2 | 0.553 | 0.041* |
| 20/ADRA1D | 4211440 | rs6116268 | 0.48 | 0.304 | 0.890 |
| 20/ADRA1D | 4215557 | rs1556832 | 0.5 | 0.327 | 0.669 |
| 20/ADRA1D | 4216663 | rs8118409 | 0.26 | 0.968 | 0.651 |
| 20/ADRA1D | 4216864 | rs4815670 | 0.45 | 0.866 | 0.322 |
| 20/ADRA1D | 4217249 | rs6133098 | 0.27 | 0.574 | 0.675 |
| 20/ADRA1D | 4222509 | rs6084670 | 0.25 | 0.453 | 0.556 |
| 20/ADRA1D | 4225394 | rs3787442 | 0.49 | 0.472 | 0.805 |
| 20/ADRA1D | 4225573 | rs6052456 | 0.19 | 0.327 | 0.139 |
| 22/COMT | 19930109 | rs737866 | 0.27 | 0.926 | 0.236 |
| 22/COMT | 19945177 | rs740603 | 0.49 | 0.947 | 0.371 |
| 22/COMT | 19951271 | rs4680 | 0.43 | 0.856 | 0.112 |
| 22/COMT | 19955692 | rs9332377 | 0.14 | 0.619 | 0.335 |
| 11/DRD4 |  | exon3 VNTR^a^ | 0.2 |  |  |

^a^For the purpose of analysis on the DRD4 exon 3 VNTR, each variable number tandem repeat was coded as 7 repeat or other such that each individual was coded as having 0, 1 or 2 copies of the 7 repeat. Position: chromosomal position under build GRCh37.p13. MAF: minor allele frequency. Slow 4 and Slow 5: p perm value. * Achieved nominal (uncorrected) significance, *α* = .05 (but not significant when corrected for the number of cognitive measures and genetic markers examined, corrected critical value *α* = .00021).
